# Determination of Carbohydrates in Lactose-Free Dairy Products to Support Food Labelling

**DOI:** 10.3390/foods10061219

**Published:** 2021-05-28

**Authors:** Sara Panseri, Radmila Pavlovic, Marta Castrica, Maria Nobile, Federica Di Cesare, Luca Maria Chiesa

**Affiliations:** Department of Health, Animal Science and Food Safety, University of Milan, Via Celoria 10, 20133 Milan, Italy; sara.panseri@unimi.it (S.P.); radmila.pavlovic1@unimi.it (R.P.); marta.castrica@unimi.it (M.C.); federica.dicesare@unimi.it (F.D.C.); luca.chiesa@unimi.it (L.M.C.)

**Keywords:** milk, cheese, lactose-free products, carbohydrates, IC-HRMS, food safety

## Abstract

Milk and its derivatives are the basis of human nutrition since childhood. Given their importance within a balanced diet, the determination of carbohydrates in milk and its derivatives is fundamental for two reasons: one of alimentary origin related to intolerances and the other one of technological origin, especially for PDO (Protected Designation of Origin) products. The dietetic approach, including lactose-free products, has a crucial role in the management of lactose intolerance, but labelling and compositional rules indicating the absence or reduced presence of lactose in food are currently not harmonised at Union level. Considering the above-mentioned issues and the absence of official methods for the determination of low sugar concentrations, we propose a new and simple IC-HRMS method to detect carbohydrates in milk and different lactose-free derivatives, which can allow the definition of concentration limits useful to characterise products suitable for specific dietary regimes.

## 1. Introduction

Milk and its derivatives are basic human foodstuffs. Even after weaning, children, as well as adults, continue to consume milk of animal origin or its derivatives as well as cheese, yogurt, butter, cream, ice cream, etc. Given their importance within a balanced diet, the determination of carbohydrates in milk and its derivatives is fundamental for two reasons: one of alimentary origin related to intolerances and the other one of technological origin.

As regards the first issue, lactose intolerance consists in the inability to correctly digest lactose, the sugar contained in a greater extent in milk, and it is caused by an insufficient presence of the enzyme lactase [1]. The disorder, which is estimated to affect at least 70% of the adult population worldwide, with different variation between regions and countries [2], can be of genetic origin, and therefore appear in childhood, or acquired in adulthood [3]. Lactase, which is normally found in the intestinal villi of the duodenum, breaks down lactose into its two simplest compounds: galactose and glucose. When lactase is not produced or is produced in an insufficient quantity, this decomposition of the sugar is impossible or, at least, deficient, resulting in the accumulation of lactose in the large intestine. Here, it begins to ferment by the bacteria present in the intestinal flora, and creates gastrointestinal symptoms, where the most frequent are diarrhoea, abdominal pain, bloating, borborygmi, constipation, and nausea. Other rare extra intestinal manifestations include headache, cognitive dysfunction, severe fatigue, and muscle and joint pain and very rare symptoms include skin lesions, ulcers, eczema, heart palpitation, urticarial, and increased micturition [1]. The treatment for intolerant individuals consists of reducing or eliminating lactose from the diet until the symptoms disappear. Consequently, the dietetic approach, including lactose-free products, has a crucial role in the management of this intolerance. To meet the dietary calcium and high-quality protein needs of intolerant individuals, the global dairy industry has developed lactose-free products using the addition of exogenous lactase, β-galactosidase, which pre-digests the lactose in milk into glucose and galactose. Furthermore, especially in newborn screening, the analysis of galactose is also very important as some individuals suffer from galactosemia, a genetic metabolic disorder [4]. In general, lactose-free products are also the best choice to avoid the major risk associated with the complete elimination of dairy products from the diet, as well as calcium deficiency compromising bone health [5]. The lactose-free dairy market, in fact, is growing very fast and is expected to reach a 9 billion turnover by 2022 and continues to outgrow dairy overall (7.3% vs. 2.3%) [6].

On the other side, from the technological point of view, carbohydrates act as anticoagulant agents and play an important role in dairy foods, not only in flavour but also in texture, colour, and viscosity [7], which are particularly important for PDO products. Moreover, the presence of high amounts of lactulose, the disaccharide containing fructose and galactose, which is not naturally found in milk but formed by lactose isomerization by high temperatures, is an important analytical indicator of the milk heat treatment it has undergone. It is therefore important to check the severity of heat treatment by analysing the lactulose content, as a chemical indicator to detect eventual frauds, capable of distinguishing UHT (Ultra High Temperature) and sterilized milks [8].

Labelling and compositional rules indicating the absence or reduced presence of lactose in food are currently not harmonised at the Union level. For the sake of clarity and consistency, the establishment of rules on the use of statements indicating the absence or reduced presence of lactose in food should be regulated under Regulation (EU, European Union) No 1169/2011 [9], taking into account the Scientific Opinion of the Authority of 10 September 2010 on lactose thresholds in lactose intolerance and galactosaemia [2,10]. Therefore, from 2016, a transitional period opened in which the indications in question are still provided on a voluntary basis, based on the conditions provided at a national level. In Italy, for example, an official note from the Ministry of Health in 2015 clarified the applicability of the EU Regulation 609/2013 and the admissibility of certain indications that can be used on labels: the indication “lactose-free” can be used for milk and dairy products with residual lactose of less than 0.1 g per 100 g or mL. There are, however, some products on the market that use the same indication with a lower threshold: less than 0.01 g per 100 g or mL; the indication “lactose-reduced”, in use only for milk and fermented milk, can be used if the lactose residue is less than 0.5 g per 100 g or 100 mL. In order to provide accurate information to consumers on the contents of lactose-free or reduced lactose-free products, the label should also include a statement, such as “The product contains glucose and galactose as a result of the breakdown of lactose” [11].

This has increased the need to develop a considerable number of methods for determining carbohydrates in milk, including less sensitive and/or older ones, such as gravimetric, polarimetric, enzymatic, or spectrophotometric analysis; however, all these methods are time-consuming because they require extensive sample preparation and cannot differentiate individual carbohydrates [12], many of which are isomers of each other, and are not suitable for measuring lactose in food products with diverse carbohydrates [4].

In the literature, a few studies (Table 1) with more specific and sensible equipment are present, such as HPLC (High Performance Liquid Chromatography) or High-Performance Anion Exchange with Pulsed Amperometric Detection (HPAE-PAD).

Considering the above-mentioned issues and the absence of official methods for the determination of low concentrations of lactose, lactulose, glucose, and galactose in dairy products, in particular in long-aged cheeses and especially in different lactose-free or reduced lactose-free products, the aim of this study was to develop a simple and sensitive analytical method based on IC-HRMS (Ion Chromatography-High Resolution Mass Spectrometry) analysis to detect carbohydrates, which can allow the definition of concentration limits that are useful to characterise products suitable for specific dietary regimes.

## 2. Materials and Methods

### 2.1. Chemicals and Reagents

All solvents used were of analytical grade, and the standards of lactose, lactulose, glucose, galactose, and glucose-d2, as an internal standard, were purchased from Merck (Darmstadt, Germany).

### 2.2. Standard Solutions

Stock standard solutions (1 mg mL^−1^) were prepared in water and kept at −20 °C, while working solutions at 1 and 0.1 µg mL^−1^ were prepared daily in water.

### 2.3. Sample Collection

A total of 48 samples were collected: 3 raw bovine milk, 3 PDO hard cheese, 3 ultra-high-temperature (UHT) semi-skimmed milk, 3 UHT whole milk, 3 UHT skimmed lactose-free milk, 3 UHT semi-skimmed lactose-free milk, 3 lactose-free whole yogurt, 3 lactose-free semi-skimmed yoghurt, 3 lactose-free cottage cheese, 3 lactose-free butter, 3 lactose-free ricotta cheese, 3 lactose-free crescenza cheese, 3 lactose-free provola cheese, 3 lactose-free mozzarella cheese, 3 lactose-free robiola cheese, and 3 lactose-free primo sale cheese samples, from different brands, were purchased from local supermarkets.

### 2.4. Sugar Extraction Protocol

One gram of grinded cheese sample or one millilitre of milk sample was added with 5 mL of 2-propanol, as extraction solvent, vortexed for 1 min, and left in an ultrasonic bath for 15 min. After centrifugation at 2500× *g*, 4 °C for 10 min, the supernatant was dried and resuspended in 500 µL of ultrapure water and centrifuged for 2 min in an Eppendorf. Afterwards, the extract was diluted 1:100 directly in a 1.5-mL autosampler vial, by adding the internal standard (50 µL from 1 mg mL^−1^ glucose-d2 solution) to a final volume of 1 mL. In product without delactosation, the internal standard could be inserted at the beginning of the procedure, because for lactose-free products, if put in, initially there was a gradual decrease in internal standard over time.

### 2.5. IC-HRMS Orbitrap Analyses

An Ionic Chromatography (IC) Dionex ICS-5000+ system (Sunnyvale, CA, USA) composed of a Dual Pump (DP), a Conductivity Detector (EG), a Detector/Chromatography Module (DC), and an Autosampler (AS-AP), coupled to a high-resolution mass spectrometer Thermo Q-Exactive Orbitrap™ (Thermo Scientific, San Jose, CA, USA), was used for the analyses. The ion chromatography analytical column was a Dionex CarboPac™ PA20 BioLC™ (Sunnyvale, CA, USA), 3 × 150 mm with a Dionex CarboPac™ PA20 Guard Column (Sunnyvale, CA, USA), 3 × 30 mm, kept at 30 °C. The eluent flow rate was 0.50 mL min^−1^ with a gradient from 12 mM KOH (aq), kept for 10 min, increased to 100 mM KOH (aq) at 15 min, held for 5 min, back to 12 mM KOH (aq) at 21 min, and maintained for 4 min, with a total run time of 25 min. The KOH eluent was neutralized using a Dionex AERS 500, 2 mm electrolytically regenerated suppressor. The injection volume was 50 μL.

**Figure 1:**
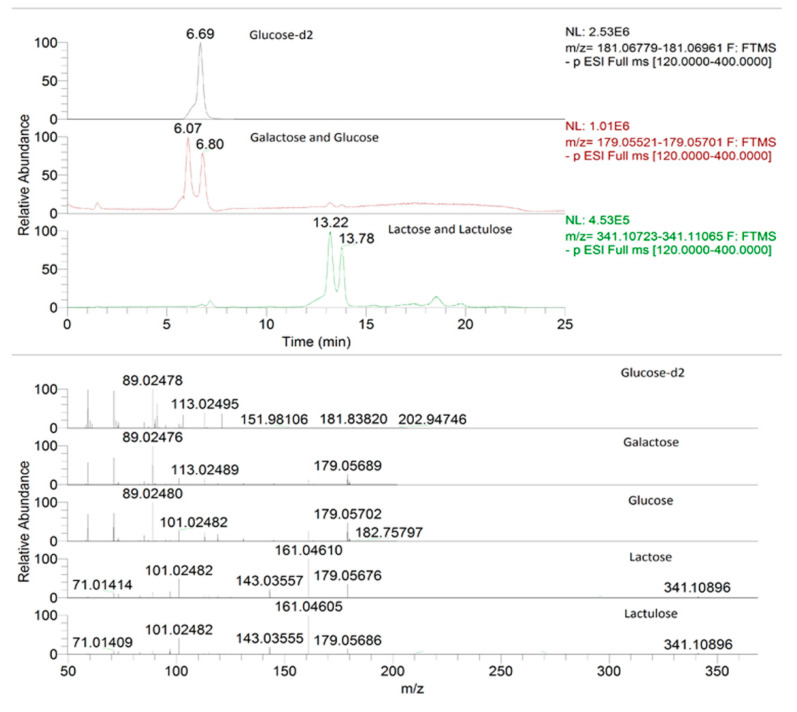
Extracted Full-MS chromatograms and mass spectra of internal standard, galactose, glucose, lactose, and lactulose at the LOD concentrations, obtained by IC-HRMS.

The MS tune parameters were capillary and vaporizer temperature set at 330 °C and 280 °C, while the electrospray voltage, operating in negative mode, was set at 3.50 kV. Sheath and auxiliary gas were at 35 and 15 arbitrary units, with an S lens RF level of 60. The Full MS-SIM (mass spectrometry-selected ion monitoring) was combined with a parallel reaction monitoring (PRM) for detecting all fragment ions in parallel, providing the confirmatory MS^2^ response, based on an inclusion list. The resolving power of Full MS-SIM was set at 70,000 full width at half maximum (FWHM); the automatic gain control (AGC) was set at 1 × 10^6^ and the maximum injection time was 100 ms. A scan range of 120–400 *m*/*z* was selected. The PRM mode operated at 17,500 FWHM, with an AGC target of 2 × 10^5^, a maximum injection time of 100 ms, an isolation window of 1 *m*/*z* and collision energy (NCE) for fragmentation set at 10 eV. In Table 2, the compounds’ formula, the parents’ exact theoretical mass, and the diagnostic transition for confirmation are presented, while in Figure 1, the extracted Full-MS chromatograms and mass spectra of internal standard, galactose, glucose, lactose, and lactulose, respectively, are reported.

ChromeleonTM (Thermo Fisher Scientific, Waltham, MA, USA) and XcaliburTM 4.3 software (Thermo Fisher Scientific, San Jose, CA, USA) were used to control the IC and HRMS system, respectively.

### 2.6. Validation of the Method

Validation was assessed according to Commission decision 657/2002/CE [20], operationally explained in our previous work [21].

In particular, for each sugar, the method performances were validated through its qualitative parameters, as well as the analyte specificity and selectivity and through its quantitative parameters, such as recovery, linearity, precision expressed as intra- and inter-day repeatability, and through the analytical limits. Detection limit (LOD) was estimated as the smallest quantity of analyte that was significantly different from the blank with a signal-to-noise ratio (S/N) of 3, while the quantification limit (LOQ) as the smallest quantity of analyte that can be measured with acceptable accuracy and precision, with a signal-to-noise ratio (S/N) of 10.

## 3. Results and Discussions

### 3.1. Method Validation Performances

The validated parameters are reported in Table 3. Good sensitivity of the method was assessed by LOD (1–10 mg kg^−1^, or 0.0001–0.001% as usually referred to for lactose-free dairy products) and LOQ values (3–30 mg kg^−1^, equivalent to 0.0003–0.003% as usually referred to for lactose-free products), especially for lactose, if compared with the detection limits reported on the label of lactose-free products (usually 0.1–0.01%) and in most cases lower than those shown in Table 1 on the literature state of art. Recoveries ranged from 92% to 94% and the precision in terms of CV% was from 6% to 7% for the intra-day validation session and from 8% to 10% for the inter-day one [20]. The linearity showed a good correlation coefficient of *R*^2^ > 0.99 for all sugars.

Moreover, as we can see from Figure 1, a satisfied chromatographic separation was achieved, considering that analytes are enantiomers amongst them, and a simple extraction protocol was applied. This underlined the selectivity of the ion chromatography and in particular the separation effectiveness of the Dionex CarboPac™ PA20 BioLC™ column for this application, which culminated in a sensitive and robust method for the combination with the powerful performances of the HRMS Orbitrap™.

### 3.2. Untargeted Analysis of Unknown Disaccharides

It is well documented that apart from lactose hydrolysis, β-galactosidases are able to catalyse a transgalactosylation reaction in which monosaccharides serve as galactosyl acceptors, yielding to so-called galactooligosaccharides (GOSs) with a different polymerisation degree and type of glycosidic bond [22]. The enzyme source and the delactosation operating conditions (lactose concentration, water activity, temperature, pH, etc.) notably influence the yield and composition of the synthesized GOS. In our analytical run, few unknown disaccharides, which do not correspond to the lactose or lactulose, were detected.

Applying HRMS detection with potential untargeted retrospective analysis, this method turned out to be highly specific for lactose and lactulose. These two compounds were well distinguished from disaccharides that resulted from the delactosation treatment, both by the retention time and mass fragmentation pattern. Especially, the detection of lactose in delactosed products was quite challenging considering that its concentration was near the LOD level and in some cases its peak appeared in the chromatographical segment (11–15 min of analytical run), where other disaccharides formed from the technological procedures used.

**Figure 2:**
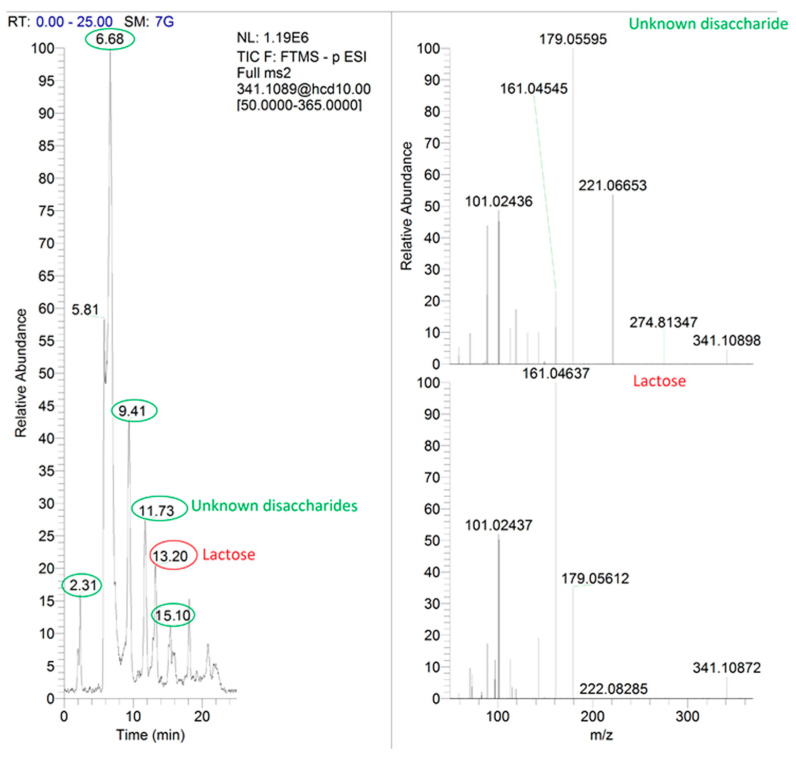
Extracted MS/MS chromatogram of unknown disaccharides (green circles) and lactose (red circle) with related mass spectra obtained from analysis of one real delactosed sample.

Particularly, in the MS/MS chromatogram extracted for the lactose anion (*m*/*z* = 341.1094), at least five signals with disaccharide fragmentation patterns were detected (Figure 2). Taking into consideration one of them (RT-11.73), close to the retention time of lactose, this unknown disaccharide gave the same fragments as lactose but with different intensities. The most important differentiating fragments were base peaks of 179.05595 and 161.04637 for unknown disaccharides and lactose, respectively (Figure 2).

Apart from lactose hydrolysis, b-galactosidases (EC 3.2.1.23) are able to catalyse a transgalactosylation reaction in which lactose or other carbohydrates in the mixture serve as galactosyl acceptors, yielding GOS with different polymerisation degrees and types of glycosidic bonds [23,24,25]. The enzyme source and the reaction operating conditions (lactose concentration, water activity, temperature, pH, etc.) notably influence the yield and composition of the synthesised GOS [26,27].In general, the GOS yield increases with increasing lactose concentration [28]. Under optimal conditions, GOS yields are between 30% and 40%(*w/w*) [29]. Apart from lactose hydrolysis, b-galactosidases (EC 3.2.1.23) are able to catalyse a transgalactosylation reaction in which lactose or other carbohydrates in the mixture serve as galactosyl acceptors, yielding GOS with different polymerisation degrees and types of glycosidic bonds [23,24,25]. The enzyme source and the reaction operating conditions (lactose concentration, water activity, temperature, pH, etc.) notably influence the yield and composition of the synthesised GOS [26,27]. In general, the GOS yield increases with increasing lactose concentration [28]. Under optimal conditions, GOS yields are between 30% and 40% (*w*/*w*) [29]. Apart from lactose hydrolysis, b-galactosidases (EC 3.2.1.23) are able to catalyse a transgalactosylation reaction in which lactose or other carbohydrates in the mixture serve as galactosyl acceptors, yielding GOS with different polymerisation degrees and types of glycosidic bonds.

### 3.3. Application to Real Samples

All the samples were analysed with the proposed and validated method, and the results are reported in Table 4. The samples were divided into the different categories of products and the average sugar content was expressed in g/100 g or g/100 mL for lactose, glucose and lactose and in mg/kg or mg/L for lactulose, as usually reported in regulations or product labels. As we can see from Table 4, through the standard deviation values, similar products from different manufacturers may differ with respect to the sugar composition due to the different process, as well as for the enzymatic hydrolysis of lactose.

In general, if we compare conventional and lactose-free products, the first one contained minor levels of free glucose and galactose, because these concentrations increase after the delactosation process, as the lactose breaks down into its constituent molecules. Since lactose hydrolysis increases the sweetness of milk, processes were developed to remove part of the lactose using chromatography and/or ultra and nano filtration operations combined with the remaining lactose hydrolysis to reach a comparable sweetness to the conventional milk [30].

The detected values were in accordance with the data reported in the literature, i.e., in the study of Ohlsson et al. [15]. As regards the lactose-free milk, we can see a slightly higher concentration of galactose and glucose in skimmed milk compared to semi-skimmed milk; in both cases, the content of lactose was less than our LOD of 0.0001 g/100 g. The slight difference in galactose and glucose in the lactose-free product with different fat concentrations could be explained by the different concentrations of lactose found in the conventional milk (raw bovine milk < whole milk, 3% fat < UHT semi-skimmed milk, 1.5% fat). This evidence also appeared in semi-skimmed lactose-free yoghurt (0.002 g/100 g) if compared with whole lactose-free yoghurt (<LOD).

Different variations in the carbohydrate content were observed amongst the different common Italian cheeses and also amongst the same categories of soft-ripened cheese, for example, for robiola and crescenza cheese. As known in the literature, hard cheeses have a very low sugar content, which in our case is lower than our LODs. Usually, in fact, most cheeses are naturally lactose free or contain very low lactose contents because, during fermentation by starter bacteria, the lactose is transformed to lactic acid in the initial stages of cheese ripening [31]. Butter is another dairy product low in lactose, because during its production, most of the water-soluble components in milk, including lactose, are removed, thereby reducing the lactose content to < 0.1% [6].

Regarding lactulose, the International Dairy Federation and the European Union have proposed it as a chemical indicator for controlling the heat processing of milk, capable of distinguishing UHT and sterilized milks. Due to the fact that UHT milks contain less lactulose than sterilized ones, both international bodies suggested 600 mg L^−1^ of lactulose as an upper limit for UHT milks [32,33]. From Table 4, lactulose was always compliant with this cut off, where the residues were found and quantitated.

## 4. Conclusions

Guiding and protecting consumers to make informed choices about the products they buy is a key objective of food safety [34,35]. The development of robust and reliable analytical methods based on innovative technologies is of paramount importance [36,37] for improving food labelling and maximising the nutritional properties of food, especially for products dedicated to susceptible consumer groups.

Lactose intolerance is a globally widespread issue affecting a very high percentage of the population, which greatly influences the habits of people with this condition. The analytical method presented in this study, with its great simplicity, reproducibility, and sensitivity, was found to be applicable to different types of milk and dairy products and can represent a valid tool for application to confirm the authenticity of the food label of lactose-free products.

## Figures and Tables

**Table 1:** State of the art about the last more specific and sensible equipment for sugar analysis.

Reference	Analytes	Matrix	Extraction Technique	Instrumental Analysis	LOD-LOQ (mg L^−1^ or mg kg^−1^)
[13]	galactose, glucose, sucrose, fructose, lactose, lactulose	1 whole milk, 1 lactose-free milk, 1 yogurt, 3 lactose-free cheese	Liquid extraction, filtration, purification by Thermo Scientific Dionex OnGuard IIA, 2.5 cc cartridge	HPAEC-PAD	MDL: 0.5–30 in water
[14]	galactose, glucose, lactose	hard cheese	Heated microwave extraction by water, sonication, filtration, extraction, filtration, SPE sulfonic acid bonding column, filtration	HPAEC-PAD	LOD: 1.4–2.5 LOQ: 2.6–4.1
[15]	lactose, glucose, galactose	milk, fermented milk and lactose-free milk products	Incubation, clarification, filtration	HPAEC-ECD	LOD: 100
[16]	lactose, lactulose	UHT milk	Defatting, clarification, dilution	UHPLC-MS/MS	LOD: 0.023–0.050 LOQ: 0.123–0.157
[17]	lactose	milk, yoghurt, chocolate drink, whipped cream, vanilla custard, cream cheese, margarine, firm cheese, whipped cream, chocolate hazelnut cream	Dilution, centrifugation, ultrafiltration.	HPAEC-PAD	LOQ: 10
[18]	lactose	raw, whole, semi-skimmed and skimmed milk	Liquid extraction, filtration	HILIC–MS/MS	LOQ: 15
[19]	lactose, lactulose	low-lactose and lactose-free milk, milk products, products containing dairy ingredients	Homogenization, dilution, biosensor assay kit	LactoSensR Amperometry Method	LOQ: 50–80

LOD (Limit of Detection); LOQ (Limit of Quantification); MDL (Method Detection Limit); HPAEC-PAD (High-Performance Anion-Exchange Chromatography with Pulsed Amperometric Detection); HPAEC-ECD (High-Performance Anion-Exchange Chromatography with Electrochemical Detector); UHPLC-MS/MS (Ultra-High-Pressure Liquid Chromatography tandem Mass Spectrometry).

**Table 2:** Carbohydrates’ formula, parents’ theoretical exact mass, and main fragments used for confirmation analysis. IS: Internal Standard.

Sugar	Formula	Parent Exact Mass [*m*/*z*]	Main Fragments [*m*/*z*]
galactose	C_6_H_12_O_6_	179.05611	89.02476; 101.02489; 113.2489
glucose	C_6_H_12_O_6_	179.05611	89.02480; 101.02482; 113.2482
lactose	C₁₂H₂₂O₁₁	341.10894	143.03557; 161.04610; 179.05676
lactulose	C₁₂H₂₂O₁₁	341.10894	143.03555; 161.04605; 179.05686
IS: Glucose-d2	C_6_H_10_D_2_O_6_	181.06867	89.02478; 101.02495; 113.2495

**Table 3:** Validation parameters of the selected carbohydrates.

Compound	LOD (mg kg^−1^)	LOQ (mg kg^−1^)	Recovery (%)	CV Intra-Day (%)	CV Inter-Day (%)	Linearity (*R*^2^)
galactose	10	30	94	6	9	0.9983
glucose	10	30	93	6	8	0.9918
lactulose	10	30	94	6	8	0.9931
lactose	1	3	92	7	10	0.9930

**Table 4:** Average sugar content (± SD) in the analysed conventional milks, PDO (Protected Designation of Origin) hard cheese, and different lactose-free products.

		g/100 g or g/100 mL			mg/kg or mg/L
Conventional Products	N° of Samples	Galactose	Glucose	Lactose	Lactulose
raw bovine milk	3	0.02 ± 0.001	0.02 ± 0.002	2.67 ± 0.53	<10
whole milk, 3% fat	3	0.01 ± 0.001	0.01 ± 0.001	4.10 ± 0.18	906.3 ± 163.4
UHT semi-skimmed milk, 1.5% fat	3	0.01 ± 0.001	0.01 ± 0.001	4.39 ± 0.09	316.3 ± 298.1
PDO hard cheese	3	<0.001	<0.001	<0.0001	<10
**Lactose-Free Products**					
UHT skimmed lactose-free milk, 0.3% fat	3	1.06 ± 0.60	1.85 ± 0.28	<0.0001	<10
UHT semi-skimmed lactose-free milk, 1.5% fat	3	0.75 ± 0.13	1.30 ± 0.34	<0.0001	<10
whole lactose-free yoghurt, 3% fat	3	0.53 ± 0.03	1.49 ± 0.24	<0.0001	350.3 ± 63.32
semi-skimmed lactose-free yoghurt, 1.5% fat	3	0.54 ± 0.04	1.19 ± 0.05	0.002	422.1 ± 71.45
lactose-free mozzarella cheese	3	0.33 ± 0.02	0.18 ± 0.02	<0.0001	<10
lactose-free robiola, soft-ripened cheese	3	0.60 ± 0.08	1.18 ± 0.05	<0.0001	122.66
lactose-free crescenza, soft-ripened cheese	3	0.44 ± 0.07	0.43 ± 0.04	0.003	<10
lactose-free cottage cheese	3	0.23 ± 0.08	0.09 ± 0.02	<0.0001	<10
lactose-free butter	3	0.02 ± 0.01	0.01 ± 0.001	<0.0001	<10
lactose-free ricotta cheese	3	0.68 ± 0.07	0.98 ± 0.06	<0.0001	<10
lactose-free provola cheese	3	0.31 ± 0.05	0.13 ± 0.02	0.002	<10
lactose-free primo sale, semi-soft cheese	3	0.41 ± 0.04	0.72 ± 0.05	<0.0001	<10
